# Genetic markers of late radiation toxicity in the era of image-guided radiotherapy: lower toxicity rates reduce the predictive value of γ-H2AX foci decay ratio in patients undergoing pelvic radiotherapy

**DOI:** 10.1186/s13014-024-02501-x

**Published:** 2024-09-02

**Authors:** Anna C. Nuijens, Arlene L. Oei, Lisa Koster, Ron A. Hoebe, Nicolaas A.P. Franken, Coen R.N. Rasch, Lukas J.A. Stalpers

**Affiliations:** 1 Department of Radiation Oncology, Amsterdam UMC location University of Amsterdam, Meibergdreef 9, Amsterdam, The Netherlands; 2 Laboratory for Experimental Oncology and Radiobiology (LEXOR), Center for Experimental and Molecular Medicine (CEMM), Amsterdam UMC location University of Amsterdam, Meibergdreef 9, Amsterdam, 1105 AZ The Netherlands; 3 Cancer Center Amsterdam, Imaging and Biomarkers, Amsterdam, The Netherlands; 4 Department of Medical Biology and Core Facility Cellular Imaging, Van Leeuwenhoek Centre for Advanced Microscopy-Academic Medical Center (LCAM-AMC), University of Amsterdam, Amsterdam, The Netherlands; 5 Department of Radiation Oncology, Leiden University Medical Center, Leiden, the Netherlands

**Keywords:** Prostate cancer, Cervical cancer, Gynecological cancer, External beam radiotherapy, Late radiation toxicity, Quality of life, Genetic predisposition, Gamma-H2AX assay

## Abstract

**Background:**

A predictive assay for late radiation toxicity would allow more personalized treatment planning, reducing the burden of toxicity for the more sensitive minority, and improving the therapeutic index for the majority. In a previous study in prostate cancer patients, the γ-H2AX foci decay ratio (γ-FDR) was the strongest predictor of late radiation toxicity. The current study aimed to validate this finding in a more varied group of patients with pelvic cancer. Additionally, the potential correlation between the γ-FDR and patient-reported outcomes was investigated.

**Methods:**

Prostate and gynecological cancer patients with ≥ 24 months of follow-up were included in the current analysis. Toxicity was evaluated by physician (CTCAE version 4) and patient (EORTC questionnaires). γ-FDRs were determined in ex vivo irradiated lymphocytes. Correlation between γ-FDR and toxicity was assessed using both linear and logistic regression analyses. The highest toxicity grade recorded during follow-up was used. The association between global quality of life and γ-FDR was tested by comparing the change in quality of life over time in patients with γ-FDR < or ≥ 3.41, a previously established threshold.

**Results:**

Eighty-eight patients were included. Physician-assessed and patient-reported cumulative grade ≥ 2 toxicity was 25% and 29%, respectively; which is much lower than in the previous cohort (i.e., 51% CTCAE grade ≥ 2). Patients with toxicity exhibited less favorable dose-volume parameters. In men, these parameters showed significant improvement compared to the previous cohort. The proportion of patients with a low γ-FDR increased with severity of toxicity, but this trend was not statistically significant. In addition, a γ-FDR < 3.41 was not correlated with the development of moderate to severe toxicity. Post-treatment decline in global quality of life was minimal, and similar for patients with γ-FDR < or ≥ 3.41.

**Conclusions:**

In the present study, the γ-H2AX foci decay ratio could not be validated as a predictor of late radiation toxicity in patients with pelvic cancer. Improved radiotherapy techniques with smaller irradiated bladder and bowel volumes have probably resulted in less toxicities. Future studies on genetic markers of toxicity should be powered on these lower incidences. We further recommend taking persistency, next to severity, into consideration.

**Supplementary Information:**

The online version contains supplementary material available at 10.1186/s13014-024-02501-x.

## Introduction

Radiotherapy plays a pivotal role in the treatment of various pelvic tumors, including localized prostate cancer and locoregionally advanced cervical cancer. After radiotherapy, approximately 30% of prostate and cervical cancer patients develop moderate or severe late radiation toxicity, though toxicity rates vary widely [1–9]. Urinary frequency, fistulae, rectal bleeding, and diarrhea are among the most common and debilitating symptoms [9, 10]. Due to the chronic tendency of several symptoms, they may negatively impact quality of life (QoL) [11–13].

Personalized treatment strategies that account for individual risk factors could potentially mitigate the development of severe late radiation toxicity following pelvic radiotherapy. With a growing population of long-term cancer survivors, the implementation of such strategies could offer substantial benefits to numerous patients. However, despite decades of research, a reliable predictive method to assess the risk of late radiation toxicity remains elusive.

Many risk factors for late radiation toxicity have been described. Most studies focus on dosimetric and clinical factors, such as dose, irradiated volume, and comorbidities. In the past decades, there has been an increasing recognition of the role of genetic predisposition as a risk factor [14–20]. Recently, we investigated the contribution of genetic predisposition, particularly compared with dose-volume factors, to the risk of late radiation toxicity in prostate cancer patients treated with external beam radiotherapy (EBRT) [19]. In this study, a functional assay measuring DNA damage repair proficiency was used. Our findings indicated that an impaired repair of DNA double-strand breaks (DSBs), reflected by a lower γ-H2AX foci decay ratio (γ-FDR), is associated with moderate to severe late radiation toxicity. DNA DSBs are the most catastrophic lesions induced by ionizing radiation. When DNA DSBs are inflicted, the phosphorylation of histone H2AX is one of the earliest events in the DNA damage repair cascade. The induction and subsequent resolution of DSBs can therefore be observed over time by the immunofluorescent detection of phosphorylated H2AX (γ-H2AX). The γ-FDR quantifies DNA DSB repair proficiency by assessing the number of γ-H2AX foci in ex vivo irradiated lymphocytes at 30 min post-irradiation, divided by the number at 24 h.

Although expected to operate independently of cancer site and normal tissue type, the γ-H2AX assay’s ability to predict late radiation toxicity via the γ-FDR metric has not yet been examined in patient groups other than those with prostate cancer. To address this knowledge gap, we initiated a new prospective study to validate our previous findings in a more varied group of pelvic cancer patients. Furthermore, recognizing the growing interest in the use of patient-reported outcome measures for the evaluation of toxicity, we extended the physician-assessed toxicity grading with patient-reported outcomes (PROs). This research holds promise for refining patient-tailored treatment strategies and improving the overall QoL for cancer survivors after pelvic radiotherapy.

## Materials and methods

### Patients

Between September 2018 and July 2021, patients diagnosed with prostate or gynecologic cancer (i.e., cervix, uterus, vagina, vulva) were accrued at the Amsterdam UMC and the Leiden UMC. This study was approved by the medical ethics committee of the Amsterdam UMC. We included the subgroup of patients that had a follow-up duration of ≥ 24 months. Eligible patients had histologically confirmed cancer and underwent EBRT with curative intent. All patients were treated with volumetric modulated arc therapy (VMAT) using 10 MV photons. Patients with a history of pelvic irradiation were excluded. Prostate cancer patients who had salvage treatment after radical prostatectomy or EBRT combined with brachytherapy were also excluded. After written informed consent was obtained, blood was drawn from all patients before initiating treatment. Lymphocytes were isolated using Ficoll-Paque density gradient separation and stored in liquid nitrogen.

### γ-H2AX foci assay

Lymphocytes were thawed and irradiated with 1 Gy γ-rays using a dedicated benchtop cell irradiator (Precision CellRad, North Branford, CT, USA) with a dose rate of approximately 1.1 Gy/min (150 kV, 5 mA). Induction and decay of radiation-induced γ-H2AX foci were determined in unstimulated G(0) cells. At 30 min and 24 h post-irradiation, lymphocytes were seeded on poly-D-lysine-coated slides and fixed in 4% paraformaldehyde. After fixation, slides were washed with PBS and were ready for immunostaining, this was performed using previously published methods [21].

### γ-H2AX foci scoring

The number of γ-H2AX foci was determined in fluorescent stack images that were made using Leica Application Suite X software. Stack images of circa 60 slices with a 200-nm interval were obtained using a Leica-DM Upright Microscope (Leica, Wetzlar Germany). These stacks were deconvolved as 1 photomicrograph using Huygens Essential software. The number of foci per nucleus was scored using Cellular Imaging (CI) software version 6.2 (MATLAB version: 9.13.0), which was developed by R.A. Hoebe at the Department of Medical Biology [22]. This software used a deep learning (DL) model that was trained on our own data with CI Annotate DL version 1.5 and CI Train DL version 2.2, which use the StarDist Deep-Learning Algorithm [22, 23]. The γ-FDR was determined by dividing the number of γ-H2AX foci 30 min post-irradiation by the number of γ-H2AX foci 24 h post-irradiation. A minimum of 100 cells per patient per condition were assessed.

### Delineations and dose-volume parameters

Delineation was carried out by a single experienced physician on CT planning images using RayStation software (version 8.99). The organs at risk (OARs) considered in this study were the bladder, anal canal, rectum, sigmoid, and bowel bag. The bladder, anal canal, and rectum were defined as previously described [19]. For the sigmoid, contouring was terminated when it was no longer visible as a traversing structure. The bowel bag was defined from the level of the most inferior bowel loop, or just above the sigmoid, whichever was more inferior. Rectum and sigmoid were excluded as part of the bag. Anteriorly, contouring was stopped at a level where no further exposure to dose was anticipated based on the treatment plan evaluation. Dose-volume histograms (DVHs) were generated for all OARs based on the clinically approved dose distribution used for treatment. The bowel bag variables were expressed as absolute volumes; for instance, the amount of milliliter receiving 30 Gy or more (bowel bag V30). Other variables were expressed as relative volumes; for example, the percentage of the bladder receiving 40 Gy or more (bladder V40). Depending on the specific analysis conducted, either the physical dose or the equieffective dose in 2 Gy per fraction (EQD2) was used. The EQD2 was calculated using an α/β ratio of 3 Gy (EQD2_3_). When OAR doses were aggregated from cervical cancer EBRT and brachytherapy, the D2cc from brachytherapy was combined with the EBRT prescribed dose converted to EQD2.

### Assessment of toxicity and QoL

Toxicity and QoL were assessed at the end of radiotherapy, and at 6, 12, 18, and 24 months after treatment. Toxicity grades were corrected for baseline conditions, QoL was also assessed at baseline. Toxicity was evaluated by both physician and patient.

PROs were assessed with the validated EORTC Core Quality of Life Questionnaire (QLQ-C30) and the prostate (-PR25), cervical (-CX24) or endometrial cancer-specific module (-EN24) [24–28]. Physician-assessed toxicity was graded according to the Common Terminology Criteria for Adverse Events (CTCAE) version 4; the first author reviewed all reported toxicities [29]. An overview of items analyzed for physician-assessed and patient-reported toxicity is presented in Table 1. Patients with residual disease after completion of treatment were not eligible for toxicity assessment. Outcomes were censored when patients were diagnosed with local, regional, or metastatic recurrence.

**Table 1:** Overview of items analyzed for physician-assessed and patient-reported toxicity

**Consistently recorded CTCAE items and EORTC questions; pairwise**	
**CTCAE item**	**Question(s)**
Abdominal pain	CX24 Q31 / EN24 Q41
Diarrhea	C30 Q17
Rectal hemorrhage	PR25 Q42 / CX24 Q33
Constipation	C30 Q16
Fecal incontinence	PR25 Q41 / CX24 Q32 / EN24 Q39
Urinary incontinence	PR25 Q36 / CX24 Q36 / EN24 Q36
Urinary tract pain	PR25 Q37 / CX24 Q35 / EN24 Q37
Urinary frequency/urgency	PR25 Q31-35 / CX24 Q34 / EN24 Q34-35
Urinary retention	CX24 Q37
Vaginal inflammation/mucositis	CX24 Q41, 43
**Consistently recorded CTCAE items and EORTC questions; non-pairwise remainder**	
**CTCAE item**	**Question(s)**
Hematuria	Feeling bloated PR25 Q43 / EN24 Q42
Gastrointestinal and urinary fistula	Passing wind EN24 Q40
Open space where any other event most likely related to radiotherapy could be documented	Urge to defecate EN24 Q38
	Limited in daily life by urinary symptoms PR25 Q39
	Limited in daily life by bowel symptoms PR25 Q40
**Baseline corrections**	
No symptoms: CTCAE grade *0* versus questionnaire outcome *1* (‘not at all’). CTCAE example: rectal hemorrhage grade 3 at time X and no rectal hemorrhage at baseline? → 3 minus 0 = 3 → Severe physician-assessed toxicity Questionnaire example: urinary incontinence outcome 4 (‘very much’) at time X and no urinary incontinence at baseline? → 4 minus 1 = 3 → Severe patient-reported toxicity	

*Abbreviations:* CTCAE = Common Terminology Criteria for Adverse Events; EORTC = European Organization for Research and Treatment of Cancer; CX24 = Cervical Cancer module; Q = question; EN24 = Endometrial Cancer module; C30 = Core Quality of Life module; PR25 = Prostate Cancer module.

### Statistical analysis

Descriptive statistics were calculated for patient characteristics. Toxicity was reported as absolute number of moderate (CTCAE grade 2 or EORTC ‘quite a bit’) and severe (CTCAE grade 3 or EORTC ‘very much’) events, and as cumulative incidence rates. The maximal graded event was considered for analyses. Numeric values were analyzed using the Student’s t-test and categorical data were assessed using the Chi-square test. Correlation between γ-FDR and toxicity was assessed using both linear and logistic regression analyses.

Concerning QoL data, per sex, the mean score, standard deviation, and standard error of the mean of all scales were calculated. Changes in global QoL before and after treatment were estimated by means of simple analysis of derived summaries. For each patient a ‘change score’ was calculated, this resulted from subtracting the new baseline Qol-score from the long-term mean QoL-score. The new baseline QoL-score was defined as the maximum score of the original baseline and the early follow-up. Long-term mean QoL-score was calculated by taking the mean of scores at 12, 18, and 24 months. The association between global QoL and γ-FDR was tested by comparing the mean change scores of patients with a γ-FDR < or ≥ 3.41, our previously established threshold [14]. A 2-sided *P*-value of ≤ 0.05 was considered statistically significant. All analyses were performed using IBM SPSS Statistics for Windows, version 28.0 [30].

## Results

### Patients

One hundred and four patients had a follow-up of ≥ 24 months. All patients met the inclusion criteria. Eleven patients were excluded because of residual or early recurrent disease; five patients were excluded because they were lost to follow-up within 3 months after the end of treatment. Eighty-eight patients were available for analysis of morbidity outcome. Baseline patient and treatment characteristics are summarized in Table 2. Comparing patients with and without CTCAE grade ≥ 2 toxicity revealed older age (*P* = 0.043) and lower KPS (*P* = 0.031) as possible clinical risk factors in men (Table S1; correction for multiple comparisons was not applied).

**Table 2:** Clinical characteristics of 88 patients with pelvic cancer treated with curative EBRT

Variable		Men (*n* = 53)		Women (*n* = 35)
		Mean (range) or *n* (%)		Mean (range) or *n* (%)
Age (y)		72.9 (57–84)		57.1 (28–81)
BMI (kg/m^2^)		26.6 (19.9–46.7)		26.9 (16.9–53.5)
KPS		93.4 (70–100)		89.4 (70–100)
Cancer type	Prostate PSA* (ng/mL) Gleason TURP	53 (100) 11.8 (2.0-140.0) 7.0 (6–10) 15 (28)	Cervix Vagina Vulva Endometrium	29 (83) 3 (8) 1 (3) 2 (6)
Histology	Adeno	53 (100)	Squamous cell Adeno Other	26 (74) 4 (12) 5 (14)
Stage (cT/FIGO)	cT1 cT2 cT3 cT4	15 (28) 25 (47) 12 (23) 1 (2)	I - IB1 - IB2 IIA1 - IIA2 - IIB IIIA - IIIB IVA - IVB Recurrence^§^	1 (3) − 8 (23) − 4 (11) 1 (3) − 3 (9) − 10 (28) 0 (0) − 4 (11) 1 (3) − 0 (0) 3 (9)
Abdominal surgery		15 (28)		13 (37)
Diabetes mellitus		8 (15)		1 (3)
Intestinal disease		6 (11)		2 (6)
Cardiovascular disease		38 (72)		11 (31)
Hypertension		26 (49)		7 (20)
Current smoking†		7 (13)		6 (17)
EQD2_x_^‡^ target	72 (20 × 3) 79 (20 × 3.2) 80 (35 × 2.2)	28 (53) 7 (13) 18 (34)	44 (25 × 1.8) 50 (25 × 2)	34 (97) 1 (3)
Concomitant treatment	Hormones	42 (79)	Brachytherapy Chemotherapy Hyperthermia	31 (89) 21 (60) 3 (9)

*Abbreviations:* EBRT = external beam radiotherapy; BMI = body mass index; KPS = Karnofsky Performance Status; PSA = prostate-specific antigen; TURP = transurethral resection of the prostate; FIGO = International Federation of Gynecology and Obstetrics; EQD2_x_ = equivalent dose in 2 Gy fractions with α/β ratio of x Gy for tumor.

^*^ Post-TURP, before radiation therapy.

^§^ After surgery.

^†^ Missing data for 26 men (49%). No missing data for other variables.

^‡^ We used an alpha/beta ratio of 3 Gy and 10 Gy for prostate and gynecologic cancer, respectively.

### Physician-assessed toxicity

CTCAE reports were available for 89% (*n* = 472) of the targeted 528 (88 patients x 6 visits) data points. No life-threatening toxicity was observed during follow-up. Grade 2 and grade 3 late radiation toxicities were recorded in 19 (22%) and 5 (6%) patients, respectively (Table 3). Women (i.e., patients irradiated for gynecologic cancer) experienced more toxicity than men (i.e., patients irradiated for prostate cancer). Specifically, 34% (12/35) of women had grade 2 toxicities versus 13% (7/53) of men, and all grade 3 events were recorded in women. This results in cumulative late grade ≥ 2 toxicities of 43% and 13% for women and men, respectively. Overall, in women more bowel than urinary grade ≥ 2 toxicity was recorded (23% vs. 14%, respectively), while in men more urinary than bowel grade ≥ 2 toxicity was recorded (9% vs. 6%, respectively). The sex difference was statistically significant for bowel toxicity (*P* = 0.017).

**Table 3:** Moderate and severe adverse events as determined according to the CTCAEv4 and EORTC

Toxicity		Physician-assessed		Patient-reported	
		Moderate (*n*** = 19)**	Severe (*n*** = 5)**	Moderate (*n*** = 21)**	Severe (*n*** = 6)**
Bowel					
	Abdominal pain	2 (50)		5 (100)	
	Diarrhea	7 (86)		3 (100)	1 (100)
	Fecal incontinence	4 (100)		2 (50)	1 (100)
	Rectal hemorrhage	1 (0)		1 (0)	1 (100)
	Constipation	1 (0)		3 (67)	
	Feeling bloated			3 (0)	1 (0)
	Proctitis^†^		1 (100)		
Urinary tract					
	Urinary tract pain	1 (0)		1 (0)	1 (0)
	Frequency	3 (0)		10 (10)	2 (0)
	Incontinence	3 (67)		3 (33)	1 (100)
	Retention	1 (0)	3 (100)	1 (100)	
Gynecologic					
	Vaginal inflammation/mucositis	4 (100)	1 (100)		2 (100)
Other					
	N. peroneus neuropathy^†^	1 (0)			
	Insufficiency fractures^†^	1 (100)			
	Unilateral lymphedema lower extremity^†^	1 (100)			

*Abbreviations:* CTCAEv4 = Common Terminology Criteria for Adverse Events version 4.0; EORTC = European Organization for Research and Treatment of Cancer.

^†^ These events were reported by physicians in the free space of our toxicity reporting system and are regarded most likely related to radiotherapy.

Data are numbers of patients with percentage of women between brackets.

Physicians: 30 grade 2 events were recorded in 19 patients and 5 grade 3 events were recorded in 5 patients. Patients: 35 grade 2 events were recorded in 21 patients and 10 grade 3 events were recorded in 6 patients.

A repeated symptom was counted as a single event.

### Patient-reported QoL

EORTC QLQ-C30 and disease specific modules were available for 85% (*n* = 449) of the targeted 528 data points. At baseline and during follow-up, women registered a lower global QoL compared to men (Table S3). Global QoL-scores were relatively constant over time; overall mean scores for women and men were 70.1 and 81.4, respectively. In general, women also had worse scores on the functioning scales and symptom scales. Trends graphs for all QoL domains are presented in Figure S1.

### Patient-reported toxicity

Questionnaire toxicity data was available for 86 of 88 patients; for one patient the baseline questionnaire was missing, the second patient did not complete any of the long-term questionnaires. During follow-up, moderate and severe toxicities were reported by 21 (24%) and 6 (7%) patients, respectively (Table 3). The cumulative rate of moderate to severee toxicity was 29% for both men (15/51) and women (10/35). Overall, women reported more moderate to severe bowel toxicity (17%) compared to urinary toxicity (9%). In contrast, men reported more moderate to severe urinary toxicity (27%) compared to bowel toxicity (10%). The sex difference was statistically significant for urinary toxicity (*P* = 0.031).

### Dose-volume parameters

The average bladder values were almost identical between the group of patients without and those with grade ≥ 2 urinary toxicity (Table S4). For the anal canal, rectum, sigmoid, and bowel bag, mean volumes were almost always higher in both men and women with grade ≥ 2 bowel toxicity compared to those without, but it was never statistically significant (Tables S5-S8). With regard to the 31 women that had EBRT and brachytherapy, mean cumulative bladder D2cc (in EQD2) was significantly higher in those with grade ≥ 2 urinary toxicity compared to those without, i.e., 78.9 Gy and 68.4 Gy, respectively (*P* = 0.032; Table S9). We did not find a correlation between late bowel toxicity and mean cumulative rectum, sigmoid, and bowel D2cc (Table S10).

**Fig. 1:**
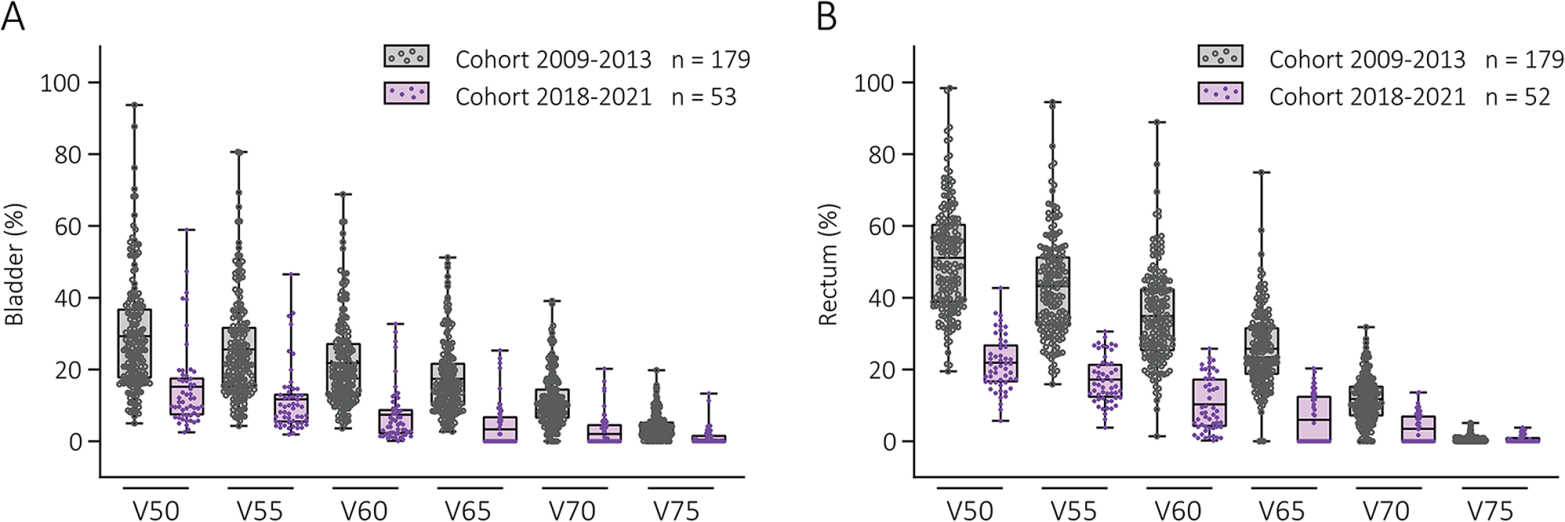
Distribution of bladder **(A)** and rectal **(B)** dose-volume parameters of prostate cancer patients from the past and current cohort. Box plots display the interquartile range, with the mean indicated, and error bars representing the full range of values. Each symbol corresponds to an individual patient. Mean V50 to V70 for the rectum and mean V50 to V75 for the bladder were significantly higher in the past cohort, with *P* < 0.001 for all parameters

Triggered by lower than expected toxicity rates, we also compared the dose-volume parameters of men from the current with the past cohort. We found that mean V50 to V70 for both rectum and bladder were significantly higher in the past cohort (*P* < 0.001; Fig. 1). For example, the mean volume of the rectum receiving 50 Gy or more (rectum V50) was 51% in the past cohort, compared to 22% in the current cohort.

### γ-H2AX foci decay ratios

**Fig. 2:**
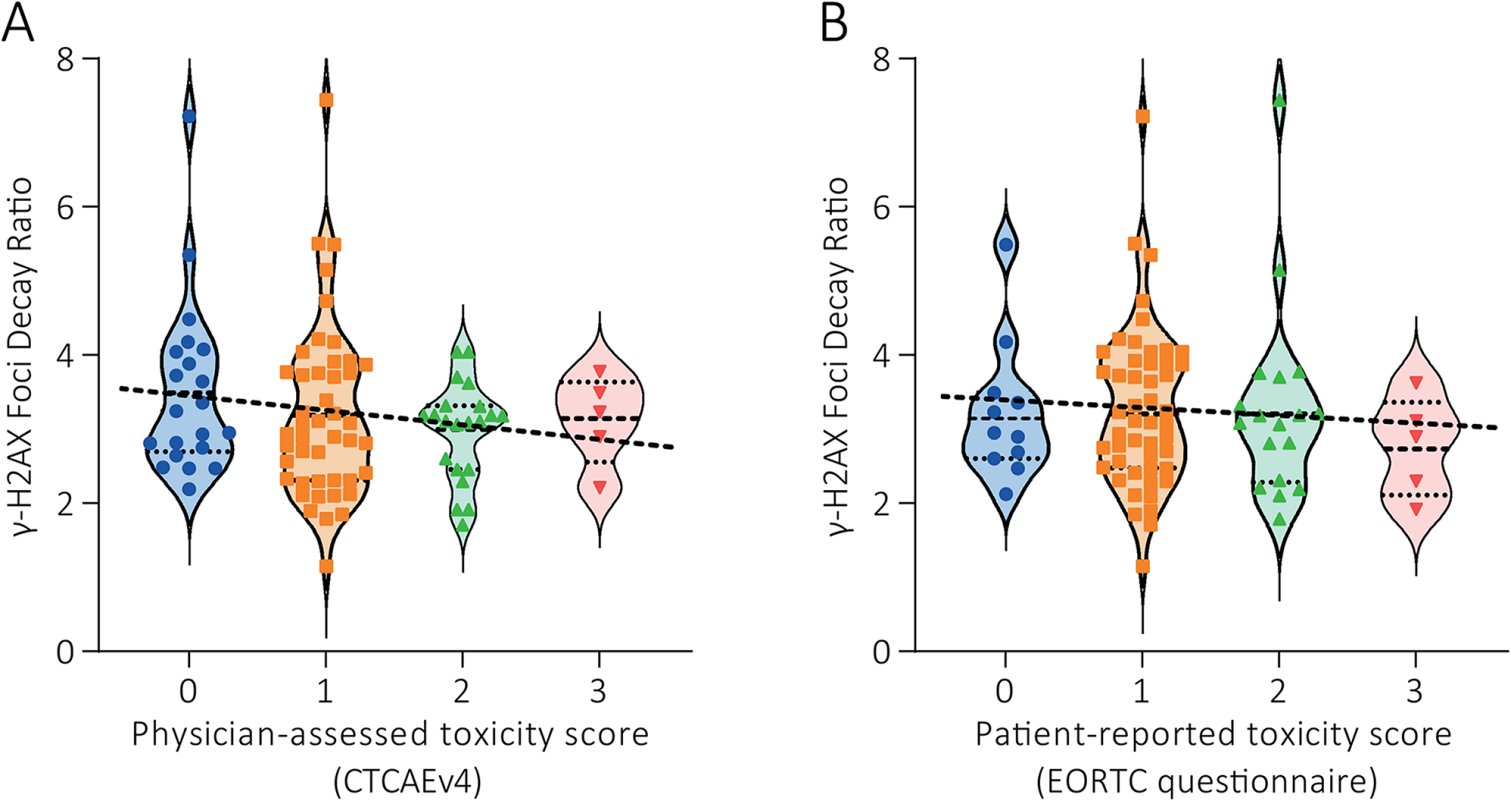
Foci decay ratios of all patients per toxicity group, patients are grouped based on their highest toxicity grade during follow-up. Foci decay ratio: number of foci at 30 min divided by number of foci at 24 h. A significant correlation between toxicity grade and foci decay ratios was not found. **(A)** Foci decay ratios versus physician-assessed toxicity score (R^2^ = 0.021; *P* = 0.184). **(B)** Foci decay ratios versus patient-reported toxicity score (R^2^ = 0.003; *P* = 0.605)

In three patients, the γ-H2AX experiment failed and did therefore not result in a γ-FDR. A significant correlation between γ-FDR and severity of physician-assessed toxicity was not found (R^2^ = 0.021; *P* = 0.184; Fig. 2A); the mean γ-FDRs per grade increase of (CTCAE) toxicity were 3.51 (SEM 0.26; *n* = 21), 3.20 (SEM 0.18; *n* = 44), 2.97 (SEM 0.17; *n* = 15), and 3.12 (SEM 0.27; *n* = 5). In addition, a γ-FDR below the threshold of 3.41 was not correlated with the development of CTCAE grade ≥ 2 toxicity (odds ratio 1.76 and *P*-value 0.329). Between γ-FDR and severity of patient-reported toxicity a correlation was also not found (R^2^ = 0.003; *P* = 0.605; Fig. 2B); the mean γ-FDRs per grade increase of toxicity were 3.22 (SEM 0.28; *n* = 11), 3.28 (SEM 0.16; *n* = 49), 3.28 (SEM 0.31; *n* = 18), and 2.77 (SEM 0.30; *n* = 5). In addition, a γ-FDR below the threshold of 3.41 was not correlated with the development of patient-reported moderate to severe toxicity (odds ratio 1.21 and *P*-value 0.712).

A γ-FDR < or ≥ 3.41, our previously established threshold, was not associated with global QoL after treatment. In both men and women, the mean change score was not significantly different between patients with γ-FDR < 3.41 and patients with γ-FDR ≥ 3.41 (Table 4).

**Table 4:** Mean global QoL change scores in men and women stratified by γ-FDR status

	γ-FDR < 3.41 (*n*)	γ-FDR ≥ 3.41 (*n*)	*P*-value
Mean change score ± SEM			
Men	-3.58 ± 1.60 (31)	-1.22 ± 3.00 (16)	0.447
Women	-2.51 ± 2.78 (21)	-5.32 ± 4.88 (12)	0.592

*Abbreviations:* QoL = quality of life; γ-FDR = γ-H2AX foci decay ratio; n = number of patients.

The change score for global QoL = long-term global QoL score (mean of scores at 12, 18, and 24 months) minus the global QoL score at redefined baseline (maximum score of original baseline and early follow-up).

## Discussion

The key to prevention of burdensome late radiation toxicities, and thus ensuring QoL after radiotherapy treatment, is accurate prediction of the risk for individuals. In this prospective study of patients irradiated for prostate or gynecologic cancer, we aimed to validate the γ-FDR as a predictive marker for late radiation toxicity. In contradiction with earlier findings, the present analysis did not demonstrate a significant correlation between the γ-FDR and severity of physician-assessed toxicity. In addition, the γ-FDR was not correlated with either patient-reported toxicity or patient-reported global QoL.

Previously, we have observed that a less efficient repair of DNA DSBs in ex vivo irradiated lymphocytes, as quantified by the γ-FDR, was an independent risk factor for the development of CTCAE grade ≥ 2 late radiation toxicity [19]. This was a prospective study in 179 prostate cancer patients curatively treated with EBRT, specifically IMRT, between 2009 and 2013. Currently, the proportion of patients with a low γ-FDR also increased with severity of toxicity, but this trend was not statistically significant. In addition, a γ-FDR below a previously established threshold of 3.41 was not correlated with the development of moderate to severe late radiation toxicity. A plausible explanation for this result could be the unexpectedly low incidence of moderate or worse toxicity. The cumulative incidences of CTCAE grade ≥ 2 late radiation toxicities in men from the previous and present study were 51% and 13%, respectively. This significant decline likely reflects the adoption of increasingly conformal treatment techniques (i.e., VMAT), which effectively reduce radiation exposure to the OARs, thereby minimizing the risk of toxicity.

Another unexpected finding was that female patients, on average, reported lower toxicity rates than physicians did. The opposite happened in men. Recently, we compared patient- and physician-reported late radiation toxicity in long-term prostate cancer survivors in detail, and found a poor agreement, with survivors reporting higher rates than physicians [31]. These results were in line with other studies wherein PROs were compared with physician-reported outcomes after radiotherapy [32, 33]. The present finding in women may be partly explained by toxicity scores that do not necessarily result from complaints, but instead result from physical and/or additional examinations. For example, three women were diagnosed with hydronephrosis accompanied by renal dysfunction, thus requiring ureteral stents (CTCAE grade 3), without experiencing any complaints.

Older age and a lower KPS seemed to be associated with toxicity in men. While some studies support these findings, consistent evidence regarding these (and other) implicated clinical risk factors remains limited in independent studies [8, 34–39]. Upon examining dosimetric factors, we found that dose-volume parameters of all gastrointestinal OARs were less favorable in patients with CTCAE grade ≥ 2 bowel toxicity, in comparison to those without. Furthermore, in women who received EBRT followed by brachytherapy, we found that the mean cumulative bladder D2cc was significantly higher in those with grade ≥ 2 urinary toxicity compared to those without.

Currently, we also studied post-treatment QoL, and its potential association with γ-FDR status. A threshold of 3.41 was previously established from data of patients with either severe or no CTCAE toxicity [14]. A γ-FDR < 3.41 was not associated with worse global QoL after treatment. This observation aligns with the understanding that QoL is multifaceted, influenced not only by toxicity but also by personal factors such as comorbidity, coping strategies, social support, and emotional well-being [31, 40–49]. In the literature, long-term QoL after EBRT for prostate cancer has been reported to be high and comparable to normative cohorts [40, 44, 50]. Schaake et al. found a statistically significant decline in several dimensions of QoL following EBRT, when compared to both baseline levels and normative data from an age-matched reference population. However, these differences were all classified as small or trivial. In addition, post-treatment global QoL did not differ from baseline [44].

The main limitation of this study was the sample size, or rather, the sample size in combination with the lower than anticipated toxicity rates. This compromised the study’s statistical power. Considering the very low incidence of late toxicity in this patient cohort, the required sample size needs to be substantially enlarged to detect a possibly very small difference in late toxicity risk by molecular or genetic markers. A post-hoc analysis assuming similar toxicity incidence in both groups (i.e., patients with y-FDR < or ≥ the threshold), showed that almost 600 patients would have been required. An other limitation applies to the duration of the follow-up period of 24 months, as toxicities can take several years to fully manifest [51, 52]. Nonetheless, other studies have shown that the majority of symptoms tend to emerge within the initial two years after radiotherapy [53, 54].

The limitations hinder a comprehensive exploration of a potential correlation between γ-FDR and the severity of toxicity. However, given the decreasing incidence of moderate or worse late radiation toxicity, the clinical importance of any found correlation between γ-FDR and maximum experienced toxicity needs to be reevaluated. In the contemporary era of increasingly conformal radiotherapy, focusing on the maximum toxicity level might not provide the most useful insights for evaluating patients well-being. Furthermore, crude incidence rates reflect the worst symptom score without considering the length of follow-up, including duration of the symptom. By disregarding (severe but) transient events, a relation to the treatment is more plausible.

The impact of toxicity on QoL has been suggested to be closely related to whether treatment-related symptoms are transient or persist over time [46, 55, 56]. Therefore, it may be more appropriate to shift our focus from maximum experienced toxicity during follow-up to persistence of toxicities. This approach is consistent with the findings of Vittrup et al., who examined late, persistent, substantial, and treatment-related symptoms (LAPERS events) in patients from the EMBRACE study. The proportion of patients with LAPERS events was substantially lower than the proportion of patients identified by crude incidence rates, thereby highlighting that the occurrence of a symptom does not necessarily equate to its persistence [46]. The observation that late toxicity can exhibit a reversible or fluctuating pattern over time has also been described in other studies [53, 57]. In line with these findings, the present study highlights the need to address the persistence of toxicity in clinical radiotherapy research.

## Conclusions

To the best of our knowledge, our study is the first to investigate the (potential) correlation between γ-FDR and the highest grade of both physician- and patient-reported toxicity. We were unable to validate the γ-FDR as a predictive marker in this relatively small sample with lower than expected toxicity rates. Improved radiotherapy techniques with smaller irradiated bladder and bowel volumes have probably resulted in these lower toxicity rates. Future studies on genetic markers of toxicity should be powered on these lower incidences. We further recommend taking persistency of toxicity, next to severity, into consideration. In conclusion, the findings underscore the need for more extensive research to fully elucidate the complexities of late radiation toxicity and its impact on patients’ well-being.

### Electronic supplementary material

Below is the link to the electronic supplementary material.

Supplementary Material 1

Supplementary Material 2

## Data Availability

The datasets generated and analyzed during the current study are not publicly available due the sensitive nature of most of the data. The datasets are available from the corresponding author on reasonable request.

## Abbreviations

γ-H2AX: Phosphorylated form of H2A histone family member X
γ-FDR: γ-H2AX foci decay ratio
CI: Cellular imaging
CTCAE: Common Terminology Criteria for Adverse Events
D2cc: Minimal dose to the most exposed 2 cc of the respective organ
DL: Deep learning
DSBs: Double-strand breaks
DVHs: Dose-volume histograms
EBRT: External beam radiotherapy
EORTC: European Organization for Research and Treatment of Cancer
EQD2: Equieffective dose in 2 Gy per fraction
OARs: Organs at risk
PROs: Patient-reported outcomes
QLQ-C30: Core Quality of Life Questionnaire
QLQ-CX24: Quality of Life Questionnaire - Cervical Cancer module
QLQ-EN24: Quality of Life Questionnaire - Endometrial Cancer module
QLQ-PR25: Quality of Life Questionnaire - Prostate Cancer module
QoL: Quality of life
V50: Volume (%) of the OAR receiving 50 Gy or more
V70: Volume (%) of the OAR receiving 70 Gy or more
