# Supplementary material for: Genetic markers of late radiation toxicity in the era of image-guided radiotherapy: lower toxicity rates reduce the predictive value of γ-H2AX foci decay ratio in patients undergoing pelvic radiotherapy
Source: Radiat Oncol. 2024 Sep 2;19:116. doi: 10.1186/s13014-024-02501-x (PMC11370123; doi:10.1186/s13014-024-02501-x)

Figure S1. Average scores of the EORTC QLQ-C30 scales according to sex. (A) Global quality of life and functional scales. (B) Symptom scales.

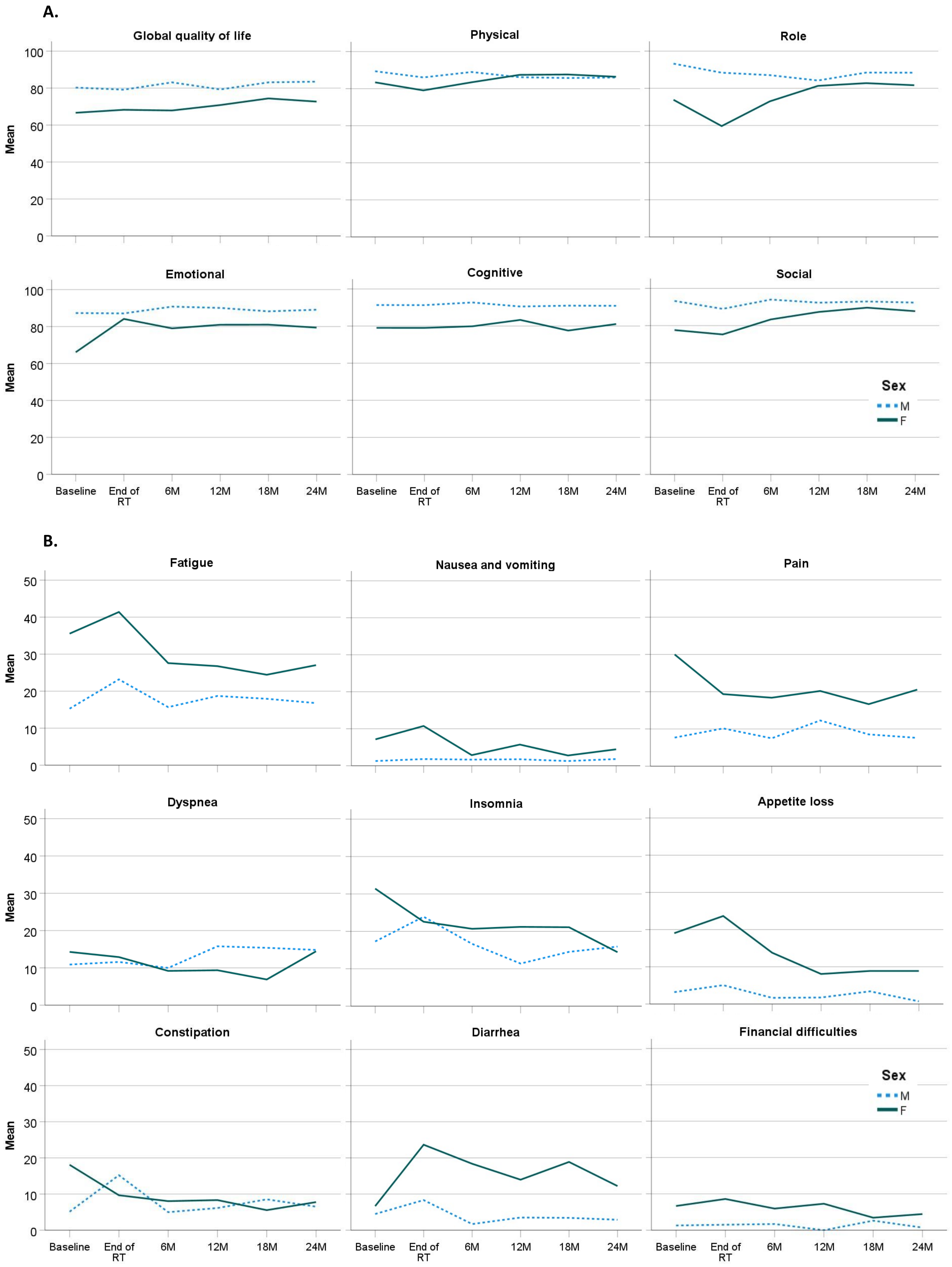

Supplement: Supplementary file 1 — Supplementary Material 1 [file 13014_2024_2501_MOESM1_ESM.pdf]
