# Supplementary material for: Genetic markers of late radiation toxicity in the era of image-guided radiotherapy: lower toxicity rates reduce the predictive value of γ-H2AX foci decay ratio in patients undergoing pelvic radiotherapy
Source: Radiat Oncol. 2024 Sep 2;19:116. doi: 10.1186/s13014-024-02501-x (PMC11370123; doi:10.1186/s13014-024-02501-x)
Supplement: Supplementary file 2 — Supplementary Material 2 [file 13014_2024_2501_MOESM2_ESM.docx]

Supplementary tables

Supplement to:
Genetic markers of late radiation toxicity in the era of image-guided radiotherapy: lower toxicity rates reduce the predictive value of γ-H2AX foci decay ratio in patients undergoing pelvic radiotherapy

Table of Contents

1. Table S1: Clinical characteristics of 53 prostate cancer patients, displayed by absence and presence of grade ≥2 toxicity (CTCAEv4)
2. Table S2: Clinical characteristics of 35 gynecologic cancer patients, displayed by absence and presence of grade ≥2 toxicity (CTCAEv4)
3. Table S3: Average scores of the EORTC QLQ-C30 scales with standard deviation (SD) and standard error of the mean (SEM) for all patients and per sex at all time points
4. Table S4: Comparison of bladder dose and grade of urinary toxicity (CTCAEv4)
5. Table S5: Comparison of rectal dose and grade of bowel toxicity (CTCAEv4)
6. Table S6: Comparison of anal canal dose and grade of bowel toxicity (CTCAEv4)
7. Table S7: Comparison of sigmoid dose and grade of bowel toxicity (CTCAEv4)
8. Table S8: Comparison of bowel bag dose and grade of bowel toxicity (CTCAEv4)
9. Table S9: Comparison of cumulative bladder D2cc and grade of urinary toxicity (CTCAEv4)
10. Table S10: Comparison of cumulative rectum/sigmoid/bowel D2cc and grade of bowel toxicity (CTCAEv4)

**Table S1.** Clinical characteristics of 53 prostate cancer patients, displayed by absence and presence of CTCAEv4 grade ≥2 toxicity

| Variable | Grade 0-1 patients  (n = 46) | Grade ≥2 patients  (n = 7) | *P* value |
| --- | --- | --- | --- |
| Age (y) | 72.2 (57 - 84) | 77.6 (71 - 81) | **0.043** |
| BMI (kg/m^2^) | 26.8 (19.9 - 46.7) | 25.5 (20.2 - 37.2) | 0.557 |
| KPS | 94.4 (70 - 100) | 87.1 (70 - 100) | **0.031** |
| Gleason score | 7.7 (6 - 10) | 7.3 (7 - 8) | 0.143 |
| PSA* (ng/mL) | 24.2 (2.0 - 140.0) | 31.0 (5.2 - 91.0) | 0.593 |
| T-stage  cT1  cT2  cT3  cT4 | 13 (28)  21 (46)  11 (24)  1 (2) | 2 (29)  4 (57)  1 (14)  0 (0) | 0.903 |
| TURP | 13 (28) | 2 (29) | 0.986 |
| Abdominal surgery | 14 (30) | 1 (14) | 0.377 |
| Diabetes mellitus | 6 (13) | 2 (29) | 0.285 |
| Intestinal disease | 6 (13) | 0 (0) | 0.310 |
| Cardiovascular disease | 34 (74) | 4 (57) | 0.359 |
| Hypertension | 24 (52) | 2 (29) | 0.245 |
| EQD2_3_ target volume  72 (20x3)  79 (20x3.2)  80 (35x2.2) | 24 (52)  6 (13)  16 (35) | 4 (57)  1 (14)  2 (29) | 0.949 |
| Hormone therapy | 37 (80) | 5 (71) | 0.584 |

*Abbreviations:* CTCAEv4 = Common Terminology Criteria for Adverse Events version 4.0; BMI = body mass index; KPS = Karnofsky Performance Status; PSA = prostate-specific antigen; TURP = transurethral resection of the prostate; EQD2_3_ = equivalent dose in 2 Gy fractions with α/β ratio of 3 Gy for tumor.
^*^ Post-TURP, before radiation therapy.
Data are mean (range) or n (%).

**Table S2.** Clinical characteristics of 35 gynecologic cancer patients, displayed by absence and presence of CTCAEv4 grade ≥2 toxicity

| Variable | Grade 0-1 patients  (n = 20) | Grade ≥2 patients  (n = 15) | *P* value |
| --- | --- | --- | --- |
| Age (y) | 60.2 (28 - 81) | 52.0 (29 - 69) | 0.101 |
| BMI (kg/m^2^) | 27.7 (20.0 - 53.5) | 25.9 (16.9 - 38.6) | 0.488 |
| KPS | 90.0 (70 - 100) | 88.7 (70 - 100) | 0.673 |
| Abdominal surgery | 9 (45) | 4 (27) | 0.267 |
| Diabetes mellitus | 1 (5) | 0 (0) | 0.380 |
| Intestinal disease | 1 (5) | 1 (7) | 0.833 |
| Cardiovascular disease | 6 (30) | 5 (33) | 0.833 |
| Hypertension | 5 (25) | 2 (13) | 0.393 |
| Current smoking | 5 (25) | 1 (7) | 0.154 |
| Histology  Squamous cell  Adeno  Other | 16 (80)  2 (10)  2 (10) | 10 (67)  2 (13)  3 (20) | 0.641 |
| FIGO stage  1 (I - IB1 - IB2)  2 (IIA1 - IIA2 - IIB)  ≥3 (IIIA - IIIB, IVA - IVB)  Recurrence^§^ | 7 (35)  9 (45)  2 (10)  2 (10) | 6 (40)  5 (33)  3 (20)  1 (7) | 0.787 |
| EQD2_10_ target volume  44,25 (25x1.8)  50 (25x2) | 19 (95)  1 (5) | 15 (100)  0 | 0.380 |
| Brachytherapy | 17 (85) | 14 (93) | 0.443 |
| Chemotherapy | 11 (55) | 10 (67) | 0.486 |
| Hyperthermia | 2 (10) | 1 (7) | 0.727 |

*Abbreviations:* CTCAEv4 = Common Terminology Criteria for Adverse Events version 4.0; BMI = body mass index; KPS = Karnofsky Performance Status; FIGO = International Federation of Gynecology and Obstetirics EQD2_10_ = equivalent dose in 2 Gy fractions with α/β ratio of 10 Gy for tumor.
^§^ After surgery.
Data are mean (range) or n (%).

**Table S3.** Average scores of the EORTC QLQ-C30 scales with standard deviation (SD) and standard error of the mean (SEM) for all patients and per sex at all time points

|  |  | **All** | | | **Men** | | | **Women** | | |
| --- | --- | --- | --- | --- | --- | --- | --- | --- | --- | --- |
| **Timepoint** | **Variable** | **Mean (n)** | **SD** | **SEM** | **Mean (n)** | **SD** | **SEM** | **Mean (n)** | **SD** | **SEM** |
| **Baseline** | Global health status/QoL | 74,9 (86) | 19,6 | 2,1 | 80,3 (52) | 16,6 | 2,3 | 66,7 (34) | 21,1 | 3,6 |
|  | Physical functioning | 87,0 (87) | 16,4 | 1,8 | 89,4 (52) | 15,7 | 2,2 | 83,4 (35) | 17,1 | 2,9 |
|  | Role functioning | 85,4 (87) | 25,1 | 2,7 | 93,3 (52) | 18,7 | 2,6 | 73,8 (35) | 28,9 | 4,9 |
|  | Emotional functioning | 78,8 (87) | 23,8 | 2,5 | 87,3 (52) | 18,7 | 2,6 | 66,1 (35) | 25,0 | 4,2 |
|  | Cognitive functioning | 86,4 (87) | 19,3 | 2.1 | 91,3 (52) | 14,6 | 2,0 | 79,0 (35) | 23,0 | 3,9 |
|  | Social functioning | 87,0 (87) | 20,2 | 2,2 | 93,3 (52) | 12,9 | 1,8 | 77,6 (35) | 25,2 | 4,3 |
|  | Fatigue | 23,4 (87) | 24,8 | 2,7 | 15,3 (52) | 20,8 | 2,9 | 35,6 (35) | 25,7 | 4,3 |
|  | Nausea/vomiting | 3,6 (87) | 9,3 | 1.0 | 1,3 (52) | 5,6 | 0,8 | 7,1 (35) | 12,3 | 2,1 |
|  | Pain | 16,7 (87) | 24,6 | 2,6 | 7,7 (52) | 18,8 | 2,6 | 30,0 (35) | 26,4 | 4,5 |
|  | Dyspnea | 12,3 (87) | 22,8 | 2,4 | 10,9 (52) | 20,6 | 2,9 | 14,3 (35) | 25,9 | 4,4 |
|  | Insomnia | 23,0 (87) | 28,0 | 3.0 | 17,3 (52) | 26,8 | 3,7 | 31,4 (35) | 27,9 | 4,7 |
|  | Appetite loss | 9,6 (87) | 19,6 | 2,1 | 3,2 (52) | 11,9 | 1,7 | 19,0 (35) | 24,6 | 4,2 |
|  | Constipation | 10,3 (87) | 20,5 | 2,2 | 5,1 (52) | 13,8 | 1,9 | 18,1 (35) | 26,0 | 4,4 |
|  | Diarrhea | 5,4 (87) | 15,1 | 1,6 | 4,5 (52) | 16,2 | 2,2 | 6,7 (35) | 13,5 | 2,3 |
|  | Financial difficulties | 3,5 (86) | 14,5 | 1,6 | 1,3 (51) | 6,5 | 0,9 | 6,7 (35) | 21,1 | 3,6 |
|  |  |  |  |  |  |  |  |  |  |  |
| **After RT** | Global health status/QoL | 74,7 (75) | 20,4 | 2,4 | 79,2 (44) | 18,1 | 2,7 | 68,3 (31) | 22,1 | 4.0 |
|  | Physical functioning | 83,3 (77) | 18,7 | 2,1 | 86,1 (46) | 16,2 | 2,4 | 79,1 (31) | 21,4 | 3,8 |
|  | Role functioning | 76,8 (77) | 28,2 | 3,2 | 88,4 (46) | 16,8 | 2,5 | 59,7 (31) | 33,0 | 5,9 |
|  | Emotional functioning | 85,9 (75) | 19,0 | 2,2 | 87,1 (44) | 21,1 | 3,2 | 84,1 (31) | 15,9 | 2,8 |
|  | Cognitive functioning | 86,2 (75) | 21,6 | 2,5 | 91,3 (44) | 21,4 | 3,2 | 79,0 (31) | 20,2 | 3,6 |
|  | Social functioning | 83,3 (75) | 24,0 | 2,8 | 89,0 (44) | 20,3 | 3,1 | 75,3 (31) | 26,8 | 4,8 |
|  | Fatigue | 30,5 (77) | 26,0 | 3,0 | 23,2 (46) | 24,2 | 3,6 | 41,4 (31) | 25,2 | 4,5 |
|  | Nausea/vomiting | 5,4 (77) | 12,2 | 1,4 | 1,8 (46) | 6,3 | 0,9 | 10,8 (31) | 16,4 | 2,9 |
|  | Pain | 13,9 (77) | 21,7 | 2,5 | 10,1 (46) | 19,4 | 2,9 | 19,4 (31) | 24,0 | 4,3 |
|  | Dyspnea | 12,1 (77) | 22,9 | 2,6 | 11,6 (46) | 22,5 | 3,3 | 12,9 (31) | 23,8 | 4,3 |
|  | Insomnia | 23,4 (77) | 29,2 | 3,3 | 23,9 (46) | 30,4 | 4,5 | 22,6 (31) | 27,7 | 5,0 |
|  | Appetite loss | 12,6 (77) | 23,6 | 2,7 | 5,1 (46) | 14,0 | 2,1 | 23,7 (31) | 30,1 | 5,4 |
|  | Constipation | 13,0 (77) | 23,7 | 2,7 | 15,2 (46) | 24,0 | 3,5 | 9,7 (31) | 23,1 | 4,1 |
|  | Diarrhea | 14,7 (75) | 24,0 | 2,8 | 8,3 (44) | 17,8 | 2,7 | 23,7 (31) | 28,8 | 5,2 |
|  | Financial difficulties | 4,4 (75) | 16,7 | 1,9 | 1,5 (44) | 7,0 | 1,1 | 8,6 (31) | 24,3 | 4,4 |
|  |  |  |  |  |  |  |  |  |  |  |
| **6M** | Global health status/QoL | 76,9 (66) | 22,8 | 2,8 | 83,1 (39) | 19,2 | 3,1 | 67,9 (27) | 24,9 | 4,8 |
|  | Physical functioning | 86,7 (69) | 18,3 | 2,2 | 89,0 (40) | 14,8 | 2,3 | 83,4 (29) | 22,2 | 4,1 |
|  | Role functioning | 81,2 (69) | 24,9 | 3,0 | 87,1 (40) | 17,9 | 2,8 | 73,0 (29) | 30,7 | 5,7 |
|  | Emotional functioning | 85,8 (68) | 19,8 | 2,4 | 90,8 (39) | 15,4 | 2,5 | 79,0 (29) | 23,2 | 4,3 |
|  | Cognitive functioning | 87,3 (68) | 18,7 | 2,3 | 92,7 (39) | 12,0 | 1,9 | 79,9 (29) | 23,3 | 4,3 |
|  | Social functioning | 89,5 (68) | 18,6 | 2,3 | 94,0 (39) | 11,8 | 1,9 | 83,3 (29) | 24,0 | 4,5 |
|  | Fatigue | 20,7 (69) | 21,6 | 2,6 | 15,7 (40) | 16,1 | 2,5 | 27,6 (29) | 26,2 | 4,9 |
|  | Nausea/vomiting | 2,2 (69) | 6,3 | 0,8 | 1,7 (40) | 6,3 | 1,0 | 2,9 (29) | 6,4 | 1,2 |
|  | Pain | 12,1 (69) | 19,4 | 2,3 | 7,5 (40) | 15,5 | 2,5 | 18,4 (29) | 22,4 | 4,2 |
|  | Dyspnea | 9,7 (69) | 17,2 | 2,1 | 10,0 (40) | 18,8 | 3,0 | 9,2 (29) | 15,2 | 2,8 |
|  | Insomnia | 18,4 (69) | 25,3 | 3,0 | 16,7 (40) | 22,6 | 3,6 | 20,7 (29) | 28,7 | 5,3 |
|  | Appetite loss | 6,8 (69) | 17,7 | 2,1 | 1,7 (40) | 7,4 | 1,2 | 13,8 (29) | 24,4 | 4,5 |
|  | Constipation | 6,3 (69) | 14,3 | 1,7 | 5,0 (40) | 12,1 | 1,9 | 8,0 (29) | 17,0 | 3,2 |
|  | Diarrhea | 8,8 (68) | 20,5 | 2,5 | 1,7 (39) | 7,4 | 1,2 | 18,4 (29) | 27,6 | 5,1 |
|  | Financial difficulties | 3,5 (67) | 13,1 | 1,6 | 1,7 (39) | 7,4 | 1,2 | 6,0 (28) | 18,3 | 3,5 |
|  |  |  |  |  |  |  |  |  |  |  |
| **12M** | Global health status/QoL | 75,5 (67) | 17,0 | 2,1 | 79,3 (37) | 16,0 | 2,6 | 70,8 (30) | 17,2 | 3,1 |
|  | Physical functioning | 86,8 (71) | 16,9 | 2,0 | 86,2 (38) | 16,8 | 2,7 | 87,5 (33) | 17,4 | 3,0 |
|  | Role functioning | 82,9 (71) | 22,9 | 2,7 | 84,2 (38) | 22,2 | 3,6 | 81,3 (33) | 23,8 | 4,2 |
|  | Emotional functioning | 85,9 (69) | 21,1 | 2,5 | 90,1 (37) | 19,4 | 3,2 | 81,0 (32) | 22,1 | 3,9 |
|  | Cognitive functioning | 87,2 (69) | 20,6 | 2,5 | 90,5 (37) | 22,1 | 3,6 | 83,3 (32) | 18,5 | 3,3 |
|  | Social functioning | 90,0 (70) | 19,1 | 2,3 | 92,3 (37) | 18,7 | 3,1 | 87,4 (33) | 19,6 | 3,4 |
|  | Fatigue | 22,5 (71) | 22,1 | 2,6 | 18,7 (38) | 20,4 | 3,3 | 26,8 (33) | 23,5 | 4,1 |
|  | Nausea/vomiting | 3,6 (70) | 8,5 | 1,0 | 1,8 (38) | 6,5 | 1,0 | 5,7 (32) | 10,0 | 1,8 |
|  | Pain | 16,0 (71) | 24,0 | 2,8 | 12,3 (38) | 23,1 | 3,8 | 20,2 (33) | 24,6 | 4,3 |
|  | Dyspnea | 12,9 (70) | 23,6 | 2,8 | 15,8 (38) | 27,7 | 4,5 | 9,4 (32) | 17,4 | 3,1 |
|  | Insomnia | 16,0 (71) | 22,4 | 2,7 | 11,4 (38) | 17,8 | 2,9 | 21,2 (33) | 26,1 | 4,5 |
|  | Appetite loss | 4,7 (71) | 13,0 | 1,5 | 1,8 (38) | 7,5 | 1,2 | 8,1 (33) | 16,7 | 2,9 |
|  | Constipation | 7,1 (70) | 14,9 | 1,8 | 6,1 (38) | 15,2 | 2,5 | 8,3 (32) | 14,7 | 2,6 |
|  | Diarrhea | 8,2 (69) | 18,4 | 2,2 | 3,5 (38) | 12,9 | 2,1 | 14,0 (31) | 22,4 | 4,0 |
|  | Financial difficulties | 3,4 (69) | 13,0 | 1,6 | 0,0 (37) | 0,0 | 0,0 | 7,3 (32) | 18,4 | 3,3 |
|  |  |  |  |  |  |  |  |  |  |  |
| **18M** | Global health status/QoL | 79,4 (67) | 17,4 | 2,1 | 83,1 (38) | 13,8 | 2,2 | 74,4 (29) | 20,5 | 3,8 |
|  | Physical functioning | 86,6 (69) | 16,7 | 2,0 | 85,8 (39) | 16,1 | 2,6 | 87,7 (30) | 17,6 | 3,2 |
|  | Role functioning | 86,0 (69) | 22,1 | 2,7 | 88,5 (39) | 19,2 | 3,1 | 82,8 (30) | 25,3 | 4,6 |
|  | Emotional functioning | 85,2 (68) | 21,1 | 2,6 | 88,2 (39) | 20,2 | 3,2 | 81,0 (29) | 21,8 | 4,1 |
|  | Cognitive functioning | 85,3 (68) | 19,9 | 2,4 | 91,0 (39) | 13,7 | 2,2 | 77,6 (29) | 24,1 | 4,5 |
|  | Social functioning | 91,5 (67) | 19,8 | 2,4 | 93,0 (38) | 17,2 | 2,8 | 89,7 (29) | 22,9 | 4,3 |
|  | Fatigue | 20,8 (69) | 22,3 | 2,7 | 17,9 (39) | 20,8 | 3,3 | 24,4 (30) | 24,0 | 4,4 |
|  | Nausea/vomiting | 1,9 (69) | 5,4 | 0,6 | 1,3 (39) | 4,5 | 0,7 | 2,8 (30) | 6,3 | 1,2 |
|  | Pain | 12,1 (69) | 19,8 | 2,4 | 8,5 (39) | 14,7 | 2,4 | 16,7 (30) | 24,4 | 4,4 |
|  | Dyspnea | 11,8 (68) | 19,8 | 2,4 | 15,4 (39) | 22,7 | 3,6 | 6,9 (29) | 13,7 | 2,6 |
|  | Insomnia | 17,4 (69) | 26,6 | 3,2 | 14,5 (39) | 23,9 | 3,8 | 21,1 (30) | 29,7 | 5,4 |
|  | Appetite loss | 5,8 (69) | 15,1 | 1,8 | 3,4 (39) | 12,8 | 2,0 | 8,9 (30) | 17,4 | 3,2 |
|  | Constipation | 7,2 (69) | 18,0 | 2,2 | 8,5 (39) | 16,6 | 2,7 | 5,6 (30) | 19,7 | 3,6 |
|  | Diarrhea | 8,8 (68) | 16,9 | 2,1 | 3,4 (39) | 10,2 | 1,6 | 16,1 (29) | 21,1 | 3,9 |
|  | Financial difficulties | 3,0 (67) | 12,6 | 1,5 | 2,6 (38) | 12,0 | 1,9 | 3,4 (29) | 13,6 | 2,5 |
|  |  |  |  |  |  |  |  |  |  |  |
| **24M** | Global health status/QoL | 79,3 (75) | 16,6 | 1,9 | 83,5 (46) | 12,6 | 1,9 | 72,7 (29) | 20,0 | 3,7 |
|  | Physical functioning | 86,2 (76) | 17,6 | 2,0 | 86,1 (46) | 16,9 | 2,5 | 86,4 (30) | 19,0 | 3,5 |
|  | Role functioning | 85,7 (76) | 21,7 | 2,5 | 88,4 (46) | 19,2 | 2,8 | 81,7 (30) | 24,9 | 4,5 |
|  | Emotional functioning | 85,3 (76) | 20,7 | 2,4 | 89,1 (46) | 21,7 | 3,2 | 79,4 (30) | 18,0 | 3,3 |
|  | Cognitive functioning | 87,1 (76) | 18,8 | 2,2 | 90,9 (46) | 15,6 | 2,3 | 81,1 (30) | 21,8 | 4,0 |
|  | Social functioning | 90,6 (76) | 20,2 | 2,3 | 92,4 (46) | 19,2 | 2,8 | 87,8 (30) | 21,9 | 4,0 |
|  | Fatigue | 20,8 (76) | 21,3 | 2,4 | 16,8 (46) | 19,7 | 2,9 | 27,0 (30) | 22,5 | 4,1 |
|  | Nausea/vomiting | 2,9 (76) | 9,6 | 1,1 | 1,8 (46) | 6,3 | 0,9 | 4,4 (30) | 13,1 | 2,4 |
|  | Pain | 12,7 (76) | 20,9 | 2,4 | 7,6 (46) | 14,4 | 2,1 | 20,6 (30) | 26,5 | 4,8 |
|  | Dyspnea | 14,7 (75) | 24,0 | 2,8 | 14,8 (45) | 25,2 | 3,8 | 14,4 (30) | 22,6 | 4,1 |
|  | Insomnia | 15,4 (76) | 24,0 | 2,8 | 15,9 (46) | 26,0 | 3,8 | 14,4 (30) | 20,9 | 3,8 |
|  | Appetite loss | 3,9 (76) | 12,1 | 1,4 | 0,7 (46) | 4,9 | 0,7 | 8,9 (30) | 17,4 | 3,2 |
|  | Constipation | 7,0 (76) | 15,7 | 1,8 | 6,5 (46) | 15,1 | 2,2 | 7,8 (30) | 16,8 | 3,1 |
|  | Diarrhea | 6,6 (76) | 17,2 | 2,0 | 2,9 (46) | 9,5 | 1,4 | 12,2 (30) | 23,9 | 4,4 |
|  | Financial difficulties | 2,2 (76) | 12,6 | 1,4 | 0,7 (46) | 4,9 | 0,7 | 4,4 (30) | 19,0 | 3,5 |

**Table S4.** Comparison of bladder dose and grade of urinary toxicity as determined according to the CTCAEv4

| Variable | Grade 0-1 patients | Grade ≥2 patients | *P* |
| --- | --- | --- | --- |
| Men | **(n = 48)** | **(n = 5)** |  |
| V30 | 34.52 ± 23.67 | 35.99 ± 23.80 | 0.895 |
| V40 | 23.55 ± 18.55 | 22.95 ± 15.43 | 0.945 |
| V50 | 15.08 ± 12.11 | 14.10 ± 8.51 | 0.862 |
| V55 | 11.50 ± 9.47 | 10.74 ± 6.53 | 0.862 |
| V60 | 7.24 ± 7.93 | 6.47 ± 5.87 | 0.834 |
| V65 | 4.32 ± 6.74 | 3.89 ± 5.34 | 0.890 |
| V70 | 3.00 ± 4.86 | 2.64 ± 3.66 | 0.875 |
| V75 | 1.36 ± 2.67 | 1.36 ± 1.94 | 1.000 |
| Women | **(n = 30)** | **(n = 5)** |  |
| V30 | 53.88 ± 18.22 | 52.26 ± 30.31 | 0.868 |
| V40 | 35.67 ± 13.47 | 36.21 ± 22.74 | 0.940 |

*Abbreviations*: CTCAEv4 = Common Terminology Criteria for Adverse Events version 4.0; V30 = volume of the bladder receiving 30 Gy or more; V40 = volume of the bladder receiving 40 Gy or more; etc.

All data represent mean volumes shown as percentage with standard deviation. Data relate to physical dose.

**Table S5.** Comparison of rectal dose and grade of bowel toxicity as determined according to the CTCAEv4

| Variable | Grade 0-1 patients | Grade ≥2 patients | *P* |
| --- | --- | --- | --- |
| Men | **(n = 49)** | **(n = 3)** |  |
| V30 | 50.47 ± 19.66 | 56.94 ± 11.24 | 0.578 |
| V40 | 32.59 ± 12.08 | 34.95 ± 10.10 | 0.742 |
| V50 | 21.63 ± 7.82 | 22.32 ± 4.11 | 0.882 |
| V55 | 16.85 ± 6.70 | 18.30 ± 3.93 | 0.714 |
| V60 | 9.86 ± 7.68 | 13.69 ± 5.00 | 0.400 |
| V65 | 5.56 ± 6.97 | 8.22 ± 7.15 | 0.523 |
| V70 | 3.16 ± 4.18 | 5.04 ± 4.39 | 0.453 |
| V75 | 0.64 ± 1.09 | 1.93 ± 1.71 | 0.059 |
| Women | **(n = 27)** | **(n = 8)** |  |
| V30 | 73.56 ± 24.24 | 86.59 ± 18.45 | 0.171 |
| V40 | 56.32 ± 23.90 | 71.92 ± 22.49 | 0.110 |

*Abbreviations*: CTCAEv4 = Common Terminology Criteria for Adverse Events version 4.0; V30 = volume of the rectum receiving 30 Gy or more; V40 = volume of the rectum receiving 40 Gy or more; etc.

All data represent mean volumes shown as percentage with standard deviation. Data relate to physical dose. No data available for one male patient with a history of rectal carcinoma treated by laparoscopic abdominoperineal resection.

**Table S6.** Comparison of anal canal dose and grade of bowel toxicity as determined according to the CTCAEv4

| Variable | Grade 0-1 patients | Grade ≥2 patients | *P* |
| --- | --- | --- | --- |
| Men | **(n = 49)** | **(n = 3)** |  |
| V5 | 66.50 ± 23.53 | 88.91 ± 17.72 | 0.112 |
| V10 | 45.91 ± 24.27 | 70.90 ± 18.50 | 0.087 |
| V25 | 18.55 ± 16.89 | 34.68 ± 14.16 | 0.113 |
| V30 | 13.91 ± 13.91 | 25.83 ± 11.11 | 0.153 |
| V35 | 10.41 ± 11.35 | 17.23 ± 7.74 | 0.312 |
| V40 | 7.90 ± 9.43 | 10.71 ± 7.20 | 0.615 |
| V65 | 0.87 ± 2.56 | 0.46 ± 0.79 | 0.786 |
| Women | **(n = 27)** | **(n = 8)** |  |
| V5 | 36.81 ± 37.43 | 64.47 ± 39.43 | 0.079 |
| V10 | 25.73 ± 34.10 | 50.00 ± 34.17 | 0.086 |
| V25 | 14.39 ± 28.36 | 23.42 ± 28.23 | 0.434 |
| V30 | 11.16 ± 23.89 | 17.44 ± 22.88 | 0.514 |
| V35 | 8.03 ± 18.05 | 13.30 ± 18.54 | 0.476 |
| V40 | 5.52 ± 13.85 | 9.67 ± 14.69 | 0.467 |

*Abbreviations*: CTCAEv4 = Common Terminology Criteria for Adverse Events version 4.0; V30 = volume of the anal canal receiving 30 Gy or more; V40 = volume of the anal canal receiving 40 Gy or more; etc.

All data represent mean volumes shown as percentage with standard deviation. Data relate to physical dose. No data available for one male patient with a history of rectal carcinoma treated by laparoscopic abdominoperineal resection.

**Table S7.** Comparison of sigmoid dose and grade of bowel toxicity as determined according to the CTCAEv4

| Variable | Grade 0-1 patients | Grade ≥2 patients | *P* |
| --- | --- | --- | --- |
| Men | **(n = 50)** | **(n = 3)** |  |
| V10 | 14.62 ± 27.49 | 33.33 ± 57.74 | 0.632 |
| V15 | 11.98 ± 23.91 | 33.30 ± 57.68 | 0.588 |
| V20 | 9.66 ± 20.14 | 29.51 ± 51.12 | 0.571 |
| Women | **(n = 27)** | **(n = 8)** |  |
| V10 | 93.39 ± 19.78 | 97.67 ± 6.17 | 0.554 |
| V15 | 88.30 ± 19.92 | 92.36 ± 9.14 | 0.583 |
| V20 | 78.27 ± 19.45 | 77.95 ± 17.56 | 0.968 |

*Abbreviations*: CTCAEv4 = Common Terminology Criteria for Adverse Events version 4.0; V10 = volume of the sigmoid receiving 10 Gy or more; V15 = volume of the sigmoid receiving 15 Gy or more; etc.

All data represent mean volumes shown as percentage with standard deviation. Data relate to physical dose.

**Table S8.** Comparison of bowel bag and grade of bowel toxicity as determined according to the CTCAEv4

| Variable | Grade 0-1 patients | Grade ≥2 patients | *P* |
| --- | --- | --- | --- |
| Men | **(n = 50)** | **(n = 3)** |  |
| V5 | 145.29 ± 437.77 | 456.53 ± 760.83 | 0.255 |
| V10 | 116.25 ± 367.10 | 390.06 ± 654.02 | 0.234 |
| V15 | 89.46 ± 295.83 | 314.22 ± 527.52 | 0.226 |
| V20 | 64.06 ± 220.02 | 215.56 ± 360.33 | 0.267 |
| V25 | 39.80 ± 138. 82 | 144.63 ± 241.29 | 0.227 |
| V30 | 21.78 ± 76.93 | 95.18 ± 159.13 | 0.509 |
| V35 | 12.31 ± 43.83 | 63.38 ± 106.38 | 0.494 |
| V40 | 7.87 ± 28.15 | 42.68 ± 72.01 | 0.491 |
| V45 | 4.33 ± 16.13 | 22.89 ± 38.78 | 0.495 |
| Women | **(n = 27)** | **(n = 8)** |  |
| V5 | 1138.76 ± 649.32 | 1243.36 ± 555.71 | 0.683 |
| V10 | 961.59 ± 537.93 | 1085.24 ± 541.84 | 0.572 |
| V15 | 734.19 ± 404.90 | 828.54 ± 420.20 | 0.570 |
| V20 | 488.77 ± 290.84 | 552.60 ± 308.30 | 0.594 |
| V25 | 298.59 ± 197.76 | 346.31 ± 212.65 | 0.559 |
| V30 | 195.21 ± 142.30 | 228.68 ± 155.58 | 0.571 |
| V35 | 140.43 ± 110.39 | 167.51 ± 125.37 | 0.558 |
| V40 | 101.38 ± 84.54 | 124.92 ± 105.23 | 0.517 |
| V45 | 42.68 ± 39.57 | 53.43 ± 56.46 | 0.545 |

*Abbreviations*: CTCAEv4 = Common Terminology Criteria for Adverse Events version 4.0; V10 = volume (mL) of the bowel bag receiving 10 Gy or more; V15 = volume of the bowel bag receiving 15 Gy or more; etc.

All data represent mean volumes shown as milliliters with standard deviation. Data relate to physical dose.

**Table S9.** Comparison of cumulative bladder D2cc (EBRT plus BT) and grade of urinary toxicity as determined according to the CTCAEv4

| Variable | Grade 0-1 patients  (n = 27) | Grade ≥2 patients (n = 4) | *P* |
| --- | --- | --- | --- |
| Cumulative bladder D2cc | 68.43 ± 8.92 | 78.91 ± 6.44 | **0.032** |

*Abbreviations*: D2cc = minimal dose delivered to the most exposed 2cc; EBRT = external beam radiotherapy; BT = brachytherapy; CTCAEv4 = Common Terminology Criteria for Adverse Events version 4.0.

Data are mean with standard deviation. Data relate to EQD2.

**Table S10.** Comparison of cumulative D2cc (EBRT plus BT) and grade of bowel toxicity as determined according to the CTCAEv4

| Variable | Grade 0-1 patients (n = 23) | Grade ≥2 patients  (n = 8) | *P* |
| --- | --- | --- | --- |
| Cumulative rectum D2cc | 57.52 ± 6.73 | 58.74 ± 6.87 | 0.664 |
| Cumulative sigmoid D2cc | 58.85 ± 9.07 | 59.98 ± 5.40 | 0.678 |
| Cumulative bowel D2cc | 57.65 ± 10.80 | 57.73 ± 6.19 | 0.980 |

*Abbreviations*: D2cc = minimal dose delivered to the most exposed 2cc; EBRT = external beam radiotherapy; BT = brachytherapy; CTCAEv4 = Common Terminology Criteria for Adverse Events version 4.0.

Data are mean with standard deviation. Data relate to EQD2.
